# Disability due to maternal common mental disorders (CMDs) as a risk factor for chronic childhood malnutrition: cross-sectional study: Incapacidade decorrente de transtornos mentais comuns (TMC) maternos como fator de risco para desnutrição crônica infantil: estudo transversal

**DOI:** 10.1590/1516-3180.2015.02342112

**Published:** 2016-05-13

**Authors:** Jorge Lopes Cavalcante-Neto, Cristiane Silvestre de Paula, Telma Maria de Menezes Toledo Florêncio, Claudio Torres de Miranda

**Affiliations:** I MSc. Assistant Professor, Universidade do Estado da Bahia (UNEB), Salvador, BA, Brazil.; II PhD. Adjunct Professor, Postgraduate Program on Developmental Disorders, Universidade Presbiteriana Mackenzie, São Paulo, SP, Brazil.; III PhD. Associate Professor II, School of Nutrition, Universidade Federal de Alagoas (UFAL), Maceió, AL, Brazil.; IV MD, PhD. Associate Professor I, School of Medicine, Universidade Federal de Alagoas (UFAL), Maceió, AL, Brazil.

**Keywords:** Child nutrition disorders, Disability evaluation, Mental health, Maternal health services, Poverty

## Abstract

**CONTEXT AND OBJECTIVE:**

The disability associated with maternal common mental disorders (CMDs) is among the possible explanations for the association between chronic childhood malnutrition and CMDs. CMDs may impair the mother’s ability to perform her role, particularly in deprived environments. The present study aimed to evaluate whether disability relating to CMDs could be part of the pathway of the association between childhood malnutrition and maternal CMDs.

**DESIGN AND SETTING:**

Cross-sectional study conducted in two institutions: one for malnourished children and another for eutrophic children living in a low-income community in the state of Alagoas, Brazil.

**METHOD:**

The cases consisted of 55 malnourished children aged from 12 to 60 months who were attending a nutritional rehabilitation center, with height-for-age z-scores < 2. The controls were 70 eutrophic children of the same age who were attending a day care center in the same area as the cases. The Self-Report Questionnaire made it possible to identify likely cases of maternal CMD. The Sheehan Disability Scale enabled evaluation of the associated disability.

**RESULTS:**

Chronic childhood malnutrition was significantly associated with maternal disability relating to CMDs (OR = 2.28; 95% CI: 1.02-5.1). The best logistic regression model using chronic childhood malnutrition as the dependent variable included the following independent variables: higher number of people living in the household; absence of the biological father from the household; and maternal disability relating to CMDs.

**CONCLUSIONS:**

If confirmed, the association between chronic childhood malnutrition and maternal disability relating to CMDs may be useful in helping to identify the causal chain between childhood malnutrition and maternal CMDs and to indicate environmental risk factors associated with chronic childhood malnutrition.

## RESUMO

**CONTEXTO E OBJETIVO:**

A incapacidade associada aos transtornos mentais comuns (TMCs) maternos está entre as explicações possíveis para a associação entre a desnutrição infantil crônica e os TMCs. Os TMCs podem comprometer a capacidade materna de desempenhar seu papel, especialmente em ambientes precários. O presente estudo objetivou avaliar se a incapacidade relacionada com TMCs pode fazer parte do processo de associação entre a desnutrição infantil e TMCs maternos.

**DESENHO E LOCALIZAÇÃO:**

Estudo transversal realizado em duas instituições, uma para crianças desnutridas e outra para crianças eutróficas vivendo em uma comunidade de baixa renda no estado de Alagoas, Brasil.

**METODOLOGIA:**

Casos foram 55 crianças desnutridas de 12 a 60 meses atendidas num centro de recuperação nutricional com escore z da idade para altura < 2. Controles foram 70 crianças eutróficas da mesma idade que frequentavam uma creche na mesma área do que os casos. O “Self-Report Questionnaire” permitiu identificar casos prováveis de TMCs maternos; a “Sheehan Disability Scale” possibilitou a avaliação de incapacidade associada.

**RESULTADOS:**

A desnutrição crônica infantil e a incapacidade maternal relacionada ao TMCs mostraram-se associados (OR = 2.28, IC 95% 1.02-5.1). O melhor modelo de regressão logística utilizando desnutrição crônica infantil como variável dependente incluiu um maior número de residentes na casa, ausência do pai biológico na residência e incapacidade materna relacionada ao TMCs como variáveis independentes.

**CONCLUSÕES:**

Se confirmada, a associação entre desnutrição crônica infantil e incapacidade materna relacionada a TMCs pode ser útil para ajudar a identificar a cadeia causal entre a desnutrição infantil e os TMCs maternos e indicar fatores de risco ambientais associados com a desnutrição crônica infantil.

## INTRODUCTION

Prospective studies have investigated the direction of associations of maternal common mental disorders (CMDs) and depression with chronic childhood malnutrition. In different cultures, these studies have shown that such disorders tend to be a risk factor for chronic childhood malnutrition.^1^^,^^2^^,^^3^

In 2011, Surkan et al.^1^ conducted a meta-analysis to investigate the association of childhood malnutrition with maternal CMDs and maternal depression worldwide. They analyzed 17 studies that included case-control, cross-sectional and cohort designs. The results showed that maternal CMDs including depression were associated with early childhood underweight and stunting.^4^^,^^5^

These studies reported that the association remained positive after adjustment for several possible confounders such as paternal and maternal education, maternal age, birth weight, infant physical health, breastfeeding practices, number of children and socioeconomic status.^1^^,^^2^^,^^6^^,^^7^^,^^8^ On the basis of the results from the meta-analysis, it was unlikely that the results were a chance finding. The adjustment for confounding factors diminished the importance of these factors, because of other explanations. The consistency of the prospective studies suggested that a temporal relationship existed, and perhaps a relationship that was less likely to be subject to information bias. The evidence for a positive association was strong.^1^^,^^2^

According to the World Health Organization (WHO) Report on Disability, the concept of disability (i.e. limitation of opportunities to take part in society on an equal level with other individuals because of social and environmental barriers) includes the impairment (loss or difference of physiological or psychological function) that may lead to this disability.^9^ Therefore, by using the concept of “disability relating to CMDs” instead of CMDs alone, the possibility of including variables relating to the environment (such as socioeconomic and cultural variables), as risk factors, can be improved. These variables may constitute an additional resource for understanding and managing the specific consequences of such mental health problems that may or may not be associated with disabilities in different environments. Furthermore, the results from a study conducted in 15 countries showed that impairments due to psychiatric illnesses may directly affect social disability (e.g. occupational role functioning, social contacts, parenting and partner role), and that the disability originating from mental disorders has a greater impact than the disability stemming from physical disorders.^10^

The concept of psychosocial care includes talking to the child, telling stories, having frequent physical contact with the child and providing a safe environment that exerts a protective effect on the child. Lack of such care may affect the child’s nutritional status negatively.^8^ The quality of mother/child interaction may indicate the quality of psychosocial care. Miranda et al. found a positive association between low interaction of the mother with the malnourished child and maternal CMDs.^11^

## OBJECTIVE

Starting from the hypothesis that maternal CMDs may be a risk factor for childhood malnutrition, the present study aimed to evaluate whether disability relating to CMDs could be part of the pathway of the association between childhood malnutrition and maternal CMDs.

## METHODS

### Design and subjects

This cross-sectional study involved a sample coming from two institutions located in the same low-income neighborhood in the city of Maceió, Brazil. The sample consisted of mother-child dyads, in which the child was between 12 and 60 months of age. Dyads were selected until the calculated minimum sample size was reached. One of the institutions was a nutritional rehabilitation center. Fifty-five children attending this institution had height-for-age z-scores ≤ -2 standard deviations (SDs), measured according to the WHO guidelines.^12^ The second institution was a day care center located in the same low-income neighborhood as the rehabilitation center. Seventy mother-eutrophic child dyads belonging to the latter institution were included in this study. These children were also aged between 12 and 60 months. Data collection took place from October 2009 to April 2010.

Before administering any data-gathering instruments, the interviewers obtained written informed consent from the mothers. The Research Ethics Committee of the Federal University of Alagoas approved this study under procedural number 012090/2009-79.

### Measurements

#### Nutritional status

Nutritional status was assessed with the aid of height-for-age (H/A) z-scores. H/A z-scores ≤ -2 SDs were taken in accordance with the WHO reference standard. For this analysis, the Anthro 2007 software was used for children aged up to five years and the Plus Anthro software for children older than five years.^12^

### Psychiatric assessment

To identify mothers with probable CMDs, the Self-Report Questionnaire (SRQ-20) was used. This consists of 20 closed questions with two alternatives for the answers (yes/no). The results from a Brazilian validation study that obtained a cutoff ≥ 8 positive responses was used to identify probable cases of CMDs (sensitivity = 83% and specificity = 80%).^13^

### Disability relating to CMDs

Mothers with an SRQ-20 score of 8 or higher were evaluated for disability by means of the Sheehan Disability Scale (SDS). SDS is a scale used not only in psychiatry but also in other chronic medical conditions. This scale has been translated into 21 languages, including Portuguese. SDS assesses three areas: occupational, social and family life. Each area is given a score from 0 to 10.^14^ In this study, SDS was considered positive whenever any kind of disability was identified. Any score higher than 0 was considered positive. On the basis of the meta-analysis study described earlier,^5^ disability was assumed to be associated with maternal CMDs and depression.

### Demographic status

The following variables were examined: demographic data, socioeconomic data, mother’s age, child’s age, maternal education, family income, number of children, number of people living in the household, work activity of both parents and presence of the child’s biological father in the household.

### Social class

Social class was defined in accordance with the five classes proposed by the Brazilian Association of Polling Companies (Associação Brasileira de Empresas de Pesquisa, ABEP). It was dichotomized by bringing together the higher classes (A, B, and C) and the lower classes (D and E). The low-income population studied here consisted of individuals belonging to classes C, D and E. Thus, we divided them as class C versus classes D + E.

### Covariables

Covariables were dichotomized based on the following *a priori* criteria: (a) children’s ages ≤ 36 months versus > 36 months, because children up to 36 months of age required more maternal attention; (b) mother’s age ≤ 29 years versus > 29 years, because of the perceived change in the social role of older women; (c) working versus non-working mothers, because of the importance of the woman as a household provider; (d) working versus non-working father or substitute, for the same reason; (e) number of rooms ≤ 3 or > 3, because of the importance of space and privacy; (f) number of people living in the household ≤ 4 or > 4, because the latter condition was considered to represent a risk of an overcrowded environment; (g) mother’s educational status, in which low was defined as < 4 and high as ≥ 4, because four years corresponds to the first phase of the Brazilian elementary school system; (h) number of children, in which low was defined as 1 and high as ≥ 2, because it was assumed that low-income families with ≥ 2 children would have additional difficulties, such as more severe financial constraints and less time to spend with each child; (i) absence of the child’s biological father from the household (yes or no), because absence of the father could be a risk factor for the child; and (j) social class, in which “E” was considered low, because it lies below the poverty line. “Family Social Economic Status (SES) and head-of-household’s educational status and occupation were taken as components of a five-level social class scale ranging from A, the highest, to E, the lowest”.^15^

The dichotomization criteria followed the cutoff points of other studies, for comparative purposes.^6^^,^^16^^,^^17^^,^^18^^,^^19^^,^^20^^,^^21^

### Statistical analyses

Odds ratios (OR) were used to compare differences between cases and controls in the bivariate analysis. To investigate the variables associated with child nutritional status and to model potential interactions, a sequence of bivariate analyses were performed on each variable, with controlling for potential confounders by all other variables (“maternal disability relating to CMDs”, “number of people living in the household”, “absence of biological father from the household” and “husband/partner in the labor market”). All the variables with P-values lower than 0.15 at this stage of the analysis were selected for the initial multivariate model. The final model retained the variables with P-values ≤ 0.05. P-values between 0.05 and 0.10 were interpreted as having borderline statistical significance.

The Hosmer-Lemeshow test was used to assess goodness-of-fit. Multicollinearity was verified by calculating the variance inflation factor; a cutoff > 10 was considered to be an indicator of collinearity. All the analyses were conducted by using the Statistical Package for the Social Sciences (SPSS), version 20.

### Sample size

The sample size planned for the study consisted of 45 cases and 45 controls. This number was based on an alpha error of 0.05, beta error of 0.2 (i.e. lower than 80%), prevalence of disability relating to depression among the mothers of eutrophic children of 20% and OR of 4.0, as measurements of clinical importance.

## RESULTS

Table 1 shows the distribution of the study variables among the mothers of malnourished and eutrophic children. Malnourished children were those with height-for-age z-scores ≤ -2 SDs, measured in accordance with the WHO guidelines. Eutrophic children were those with z-scores > -1 and < 1. Only the disability associated with CMDs was investigated. Mothers of malnourished children presented twice as much chance of presenting disability as did mothers of eutrophic children (OR = 2.28; 95% CI = 1.02-5.1). The cases and controls were similar with regard to most SES factors, including the mother’s age, mother in the labor market, mother’s educational status, number of rooms in the household, number of children in the household and social class. On the other hand, in the families with malnourished children, the percentage with a working father or substitute was lower than in the families of the controls (50% versus 70%; OR = 0.44; 95% CI = 0.21-0.93). At the same time, the number of people living in the household was higher in families with malnourished children than in families with eutrophic children (OR = 2.67; 95% CI = 1.17-6.11). Finally, absence of the biological father from the household was more common in families with malnourished children than in families with eutrophic children (OR = 3.14; 95% CI = 1.12-8.76).

**Table 1.**
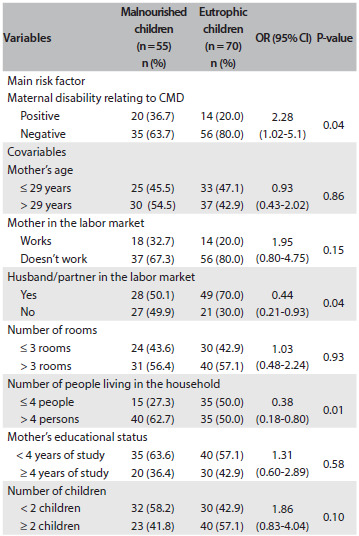
Association between child nutritional status (cases and controls) and disability associated with maternal common mental disorders (CMDs) and selected covariables

*n = 98 instead of 125; OR = odds ratio; CI = confidence interval.

Construction of a logistic regression model to include the four factors singly associated with childhood malnutrition in Table 1 helped in ascertaining whether the combination of disability associated with maternal CMDs and sociodemographic factors had any effect on the risk that a child would be malnourished. In constructing the logistic regression, the following independent variables were included: maternal disability relating to CMDs; husband/partner in the labor market; number of people living in the household; absence of the child’s father from the household; and number of children. The final and most parsimonious model identified three independent correlates (Table 2): (1) children with malnutrition were more than twice as likely to have a mother presenting disability associated with maternal CMDs (OR = 2.446; 95% CI = 1.059-5.649; P = 0.036); (2) children whose biological father was absent from the household were twice as likely to have a mother with disability associated with childhood malnutrition (OR = 2.097; 95% CI = 0.958-4.589; P = 0.064); and (3) smaller numbers of people living in the household had a protective effect (OR = 0.778; 95% CI = 0.631-0.959; P = 0.018).

**Table 2.**
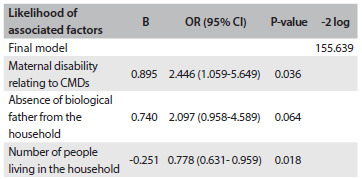
Initial and final multiple logistic regression model on factors associated with childhood malnutrition (n = 125)

CMDs = common mental disorders; OR = odds ratio; CI = confidence interval.

## DISCUSSION

The present study found that three factors were associated with childhood malnutrition: disability associated with maternal CMDs; higher numbers of people living in the household, which points towards overcrowding; and absence of the biological father, which suggests that the lack of social support matters.

To the best of our knowledge, no other studies have addressed the association between childhood malnutrition and disability relating to the mother’s mental health. The cohort study of Rahman et al. examined the association between maternal depression and infant malnutrition and assessed maternal disability among both depressed and non-depressed mothers.^2^ These authors reported that depressed mothers had higher scores for disability than did non-depressed mothers. They also stated that the disability presented by these mothers could prevent them from taking proper care of their children, and therefore it constituted a risk factor for impairment of the infants’ nutritional status.^2^ A review study on the consequences of maternal CMDs for child development (including nutritional status) listed maternal depression, social support and overcrowding as risk factors. However, this study did not specify the maternal disability.^6^

The findings from the present investigation are in agreement with those from previous studies: higher numbers of people living in the same household (overcrowding), in comparison with controls constituted another variable that was associated with childhood malnutrition. In a cross-sectional study conducted in Pakistan, Shah et al. found that children younger than three years of age who were living in more crowded households were more likely to present stunting.^20^ Similarly, in a case control study on Mexican children (aged 0 to 2 years), Sandoval-Priego et al. found that overcrowding was one of the risk factors for chronic malnutrition (in a logistic regression model).^21^ Absence of the biological father from the household was the third variable in the logistic regression that was associated with childhood malnutrition. Similarly, another case-control study conducted in an urban area in southeastern Brazil found a positive association between childhood malnutrition and the absence of the biological father, regardless of per capita income and maternal educational status. The authors of that study hypothesized that the presence of the biological father was the most important component of social support for the mother in that environment. They elaborated on this topic by stating, “with the progressive extinction of the extended family, the father may be considered to be the mother’s main supplier of emotional and material support in caring for the child”.^22^

The present study presents some limitations. Although the SDS is an instrument that can provide quantitative disability measurements, disability was only classified as present or absent in this study, because of the small sample size. The cross-sectional data collection procedure, which was conducted for operational reasons, and also the dichotomization of continuous variables, formed limitations. The study would have benefited from a larger number of available cases of stunted children.

The association between chronic childhood malnutrition and maternal CMD-related disability may help to identify the causal chain between childhood malnutrition and maternal CMDs and to point out social and environmental risk factors associated with chronic childhood malnutrition. According to the “World report on disability”, this disability not only involves mental impairment, but also includes social and environmental barriers.^9^ Therefore, adequate management of CMD-related disability might help to overcome internal and external factors that are linked to the mother’s lack of autonomy.^23^

## CONCLUSIONS

Overall, it is possible to assume that the disability associated with maternal CMDs may vary according to the environment in which the person lives. In addition to the relationship with CMDs, this disability also relates to the interaction between the subject and the environment. This interaction will be important for defining the social support that should be made available.

Further prospective studies are necessary, in order to ascertain the association between chronic childhood malnutrition and maternal disability relating to CMDs.

## References

[1] Surkan PJ, Kennedy CE, Hurley KM, Black MM (2011). Maternal depression and early childhood growth in developing countries: systematic review and meta-analysis. Bull World Health Organ.

[2] Rahman A, Iqbal Z, Bunn J, Lovel H, Harrington R (2004). Impact of maternal depression on infant nutritional status and illness: a cohort study. Arch Gen Psychiatry.

[3] Patel V, DeSouza N, Rodrigues M (2003). Postnatal depression and infant growth and development in low income countries: a cohort study from Goa, India. Arch Dis Child.

[4] de Miranda CT, Turecki G, Mari Jde J (1996). Mental health of the mothers of malnourished children. Int J Epidemiol.

[5] Rahman A, Lovel H, Bunn J, Iqbal Z, Harrington J (2004). Mothers’ mental health and infant growth: a case-control study from Rawalpindi, Pakistan. Child Care Health Dev.

[6] Santos DS, Santos DN, Silva Rde C, Hasselmann MH, Barreto ML (2011). Maternal common mental disorders and malnutrition in children: a case-control study. Soc Psychiatry Psychiatr Epidemiol.

[7] Surkan PJ, Kawachi I, Ryan LM (2008). Maternal depressive symptoms, parenting self-efficacy, and child growth. Am J Public Health.

[8] Carvalhaes MA, Benício MH (2006). Desnutrição no segundo ano de vida e cuidado psicossocial: estudo caso-controle em área urbana do Sudeste brasileiro [Malnutrition in the second year of life and psychosocial care: a case-control study in an urban area of Southeast Brazil]. Cad Saude Publica.

[9] World Health Organization (2011). World report on disability.

[10] Ormel J, VonKorff M, Ustun TB (1994). Common mental disorders and disability across cultures. Results from the WHO Collaborative Study on Psychological Problems in General Health Care. JAMA.

[11] Miranda CT, Paula CS, Santos L (2000). Association between mother-child interaction and mental health among mothers of malnourished children. J Trop Pediatr.

[12] World Health Organization Child growth standards. WHO Anthro (version 3.2.2, January 2011) and macros.

[13] Mari JJ, Williams P (1986). A validity study of a psychiatric screening questionnaire (SRQ-20) in primary care in the city of Sao Paulo. Br J Psychiatry.

[14] Sheehan DV, Harnnet-Sheehan K, Raj BA (1996). The measurement of disability. Int Clin Psychopharmacol.

[15] Associação Brasileira de Empresas de Pesquisa (2003). Critério de Classificação Econômica Brasil (CCEB 2003 - Base LSE 2000).

[16] Almeida-Filho N, Lessa I, Magalhães L (2004). Social inequality and depressive disorders in Bahia, Brazil: interactions of gender, ethnicity, and social class. Soc Sci Med.

[17] Parsons CE, Young KS, Rochat TJ, Kringelbach ML, Stein A (2012). Postnatal depression and its effects on child development: a review of evidence from low-and middle-income countries. Br Med Bull.

[18] Harpham T, Huttly S, De Silva MJ, Abramsky T (2005). Maternal mental health and child nutritional status in four developing countries. J Epidemiol Community Health.

[19] Rahman A, Lovel H, Bunn J, Iqbal Z, Harrington R (2004). Mothers’ mental health and infant growth: a case-control study from Rawalpindi, Pakistan. Child Care Health Dev.

[20] Shah SM, Selwyn BJ, Luby S, Merchant A, Bano R (2003). Prevalence and correlates of stunting among children in rural Pakistan. Pediatr Int.

[21] Sandoval-Priego AA, Reyes-Morales H, Pérez-Cuevas R, Abrego-Blas R, Orrico-Torres ES (2002). Estrategias familiares de vida y su relación con desnutrición en niños menores de dos años [Family life strategies associated with malnutrition in children aged under two years]. Salud Publica Mex.

[22] de B L Carvalhaes MA, D’Aquino Benício MH, Barros AJ (2005). Social support and infant malnutrition: a case-control study in an urban area of Southeastern Brazil. Br J Nutr.

[23] Carlson GJ, Kordas K, Murray-Kolb LE (2015). Associations between women’s autonomy and child nutritional status: a review of the literature. Matern Child Nutr.

